# The Bittersweet Beat: Diabetes Complications

**DOI:** 10.3390/jcm12124018

**Published:** 2023-06-13

**Authors:** Joana Rossell, Marina Idalia Rojo-López, Josep Julve, Didac Mauricio

**Affiliations:** 1 Institut d’Investigació Biomèdica de l’Hospital de la Santa Creu i Sant Pau (IIB-Sant Pau), 08041 Barcelona, Spain; jrossell@santpau.cat (J.R.); jjulve@santpau.cat (J.J.); 2 CIBER de Diabetes y Enfermedades Metabólicas Asociadas (CIBERDEM), 08041 Barcelona, Spain; 3 Department of Endocrinology & Nutrition, Hospital de la Santa Creu i Sant Pau, 08041 Barcelona, Spain; 4 Faculty of Medicine, University of Vic/Central University of Catalonia (UVIC/UCC), 08500 Vic, Spain

In this Editorial, we are focusing on a selection of articles recently published in the *Journal of Clinical Medicine* dealing with relevant aspects of cardiometabolic complications of diabetes mellitus. Epidemiological studies have yielded valuable insights into the complex relationship between diabetes and cardiovascular disease (CVD). Individuals with diabetes, both type 1 (T1D) and type 2 (T2D), face an increased risk of developing CVD, including peripheral artery disease, coronary artery disease, stroke, and heart failure (HF). Nonetheless, the pathophysiological mechanisms behind CVD differ between T1D and T2D [1]. Subjects with T1D have a nearly three times higher mortality risk than the general population, primarily due to CVD. They also face increased CVD risk, increased risk of hospitalization for CVD, and a worsened HF prognosis [1,2]. These findings underscore the need for comprehensive cardiovascular risk assessment, monitoring, and management strategies in people with diabetes, especially targeted for subjects with T1D, as underlined in recent publications [1,2].

Regarding the evaluation of cardiovascular risk, the use of traditional risk factors—hyperglycemia, dyslipidemia, and hypertension—does not fully account for the higher cardiovascular risk of subjects with T1D [1]. T1D subjects with fair glycemic control still harbor a higher risk of developing CVD; notably, hypoglycemia or peaks of hyperglycemia contribute to CVD risk. Further, alterations to the composition and functionality of lipoproteins are also involved and are independent from glycemic control, contributing to the complex pathophysiology of atherosclerotic CVD in subjects with T1D [2]. Current recommendations for preventing CVD in subjects with T1D are based on studies mainly performed in T2D, although the mechanisms leading to the development of CVD in both diabetes types may differ in part [2,3]. Therefore, specific guidelines may be needed to improve cardiovascular management in T1D. Different studies, including one recently performed in the Mediterranean region [3], underscore the suboptimal management of cardiovascular disease in these subjects. In particular, this study showed that a significant propor-tion of T1D subjects, many of them smokers, did not achieve adequate lipid and blood pressure control, even in the presence of CVD [3].

The findings of the above-mentioned study prove that microvascular and macrovascular disease still have a high prevalence in people with T1D [3]. It is well-known that in diabetes mellitus an acceleration of vascular aging occurs [4]. In this sense, a recent review developed the concept that the arterial wall of large arteries should be considered as a target organ based on the understanding that the stiffening of the arterial wall plays a key role in the development and accelerated progression of both macrovascular and microvascular complications in individuals with diabetes [4]. They concluded that the determination of arterial stiffness should be considered as a measure of cardiovascular injury in diabetes.

In cardiovascular research, the main focus has traditionally been the study of microvascular and macrovascular mechanisms as the major contributors to adverse vascular outcomes; however, recent evidence also suggests a role for nonvascular mechanisms as a relevant component of myocardial dysfunction in diabetes. The diabetic milieu is metabolically complex, characterized by alterations in glucose metabolism, lipids, and insulin signaling, which define the new concept of “cardiogenic diabetes” presented by Triposkiadis et al. in their review on HF in diabetes [5]. Together, these alterations can act as cardiotoxic boosters, which are particularly important in new-onset T2D subjects with HF, a common complication in diabetes. In T1D, HF typically arises due to dysregulated immune responses linked in part to high glycated hemoglobin, while in most cases of T2D, it occurs against a background of overweight or obesity. They hypothesized that such cardiogenicity may be, at least partly, attributed to nonvascular mechanisms in diabetic subjects [5]. The increased availability of glucose drives tissue glucotoxicity, which is another recognized risk factor for CVD. Whether cardiovascular remodeling and dysfunction due to glycation and glycosylation mechanisms lead to CVD in diabetic subjects was addressed in an excellent recent review by Dozio E et al. [6]. In this paper, the authors gained insight into the contribution of these mechanisms to cardiovascular remodeling and its relationship with CVD; they specifically revised the role of advanced glycation end products and O-linked-N-acetylglucosaminylation processes.

The burden of other serious complications, i.e., NAFLD and diabetic kidney disease (DKD), is also higher in subjects with diabetes, suggesting that the metabolic insult of hyperglycemia also has a strong impact on their development and progression. As such, beyond the cardiovascular system, another interesting point to note is the impact of metabolic insults due to deficient insulin signaling over other systems and tissues, including the liver (i.e., NAFLD) [7,8]. Although the notion that NAFLD is an independent risk factor for CVD is well known, Niederseer et al. produced an excellent comprehensive review developing the concept that the close relationship between NAFLD and CVD may be bidirectional [7]. Likewise, in another review Perdomo et al. further elaborated on the pathophysiology of chronic kidney disease (CKD) as a risk factor for CVD, and the fact that CKD also shares common pathophysiological features with NAFLD in subjects with T2D, including enhanced oxidative stress, inflammation, and the activated renin-angiotensin-aldosterone system; however, genetic factors, gut dysbiosis, and environmental factors also contribute to the development and progression of NAFLD, CVD, and CKD [8]. Overall, these data highlight the need for the early diagnosis and treatment of NAFLD as essential tools to reduce the burden of CKD and CVD.

Both persistent hyperglycemia and insulin resistance also disturb the physiological balance between clotting and fibrinolysis [9]. The resulting prothrombotic state may also be enhanced by other coexisting factors in diabetes, i.e., hypoglycemia, obesity, and dyslipidemia; they all further contribute to increased coagulation disorders in subjects with diabetes mellitus. In line with this, disease conditions related to the altered prothrombotic state are frequently linked to a higher risk of stroke. Progress has been made in stroke prevention and early diagnosis among individuals with diabetes mellitus, primarily through the improved management of these risk factors [10]. Despite this, the current guidelines are devoid of recommendations on the optimal antithrombotic treatment for diabetic subjects [9].

In diabetes, among other risk factors, several behavioral and lifestyle factors, including smoking, sedentarism, and poor nutritional habits, can contribute to the development of chronic complications. Interestingly, an unhealthy lifestyle, involving, at least in part, changes to the composition of intestinal microbiota, may worsen metabolic syndrome-related complications, and hence increase the risk for CVD in diabetic subjects [7]. In this regard, one of the selected studies examined the potential role of the circulating gut microbiota-derived metabolites trimethylamine N-oxide (TMAO) and branched-chain amino acids (BCAA) as novel risk markers for cardiovascular mortality in subjects with T2D [11]. Remarkably, higher plasmatic TMAO concentrations, but not those of BCAA, were associated with an increased risk of cardiovascular mortality in T2D subjects after adjusting for other clinical and biochemical risk markers. These findings suggest that TMAO may be a useful biomarker for identifying subjects with T2D who are at a higher risk of cardiovascular mortality.

Optimal medical therapy for diabetes is crucial to achieve the adequate optimization of the metabolic status and to prevent the development of its related comorbidities. The first line of action includes a dietary intervention. In the review by Katsi et al. [12], the authors dealt with the importance of the alignment of the timing of food intake to the metabolic circadian rhythms, also termed chrononutrition, and its potential benefits on cardiovascular health. Specific chrononutrition-based dietary therapies studied include intermittent fasting, which encompasses both a period of fasting and a period of regular, unrestricted eating, and time-restricted eating, which offers a way to reduce daily calorie intake by eating within a specific time frame (less than 10 h per day), and is followed by fasting for the remaining hours. They reviewed the different studies that show fairly consistent reductions in blood pressure, adiposity, glucose levels, and triglycerides when implementing these approaches. However, more extensive and consistent studies on the impact of chrononutrition on cardiovascular outcomes are required.

In addition to lifestyle measures, there are new tools to prevent chronic complications of diabetes, i.e., newer classes of glucose-lowering medications, such as glucagon-like peptide-1 receptor agonists and sodium glucose co-transporter inhibitors (SGTL2i). In this context, the use of these agents has been also proven to exert direct anticoagulation effects in subjects with diabetes [9]. In the same line, the positive impact of treatments that are commonly used in the management of HF (e.g., sacubitril/valsartan) and the benefits of SGTL2i on the development and progression of cardiomyopathy in subjects with diabetes have been excellently reviewed [5]. SGLT2i has been shown to reduce the occurrence of major adverse cardiovascular events and hospitalizations for HF in patients with T2D [13]. While clinical trial data may have shown less significant results for atherosclerotic cardiovascular events, such as heart attack and stroke, when compared with HF, real-world evidence suggests benefits in those areas as well [13]. The cardioprotective mechanisms of SGLT2 inhibitors may also involve the improvement of vascular endothelial cell function, the reduction of oxidative stress, the inhibition of inflammation, and the regulation of autophagy, all of which could contribute to the prevention of atherosclerosis progression [13].

In conclusion, CVD is one of the leading causes of mortality in both T1D and T2D, although pathways leading to its development in both types of diabetes may not always coincide. Further dedicated studies on CVD in T1D patients are needed to improve its management. Additionally, further progress in the field of cardiometabolic complications of diabetes has already been introduced in the current management of these conditions. However, more translational and clinical research is also warranted to increase our knowledge and, thus, the clinical management of these complications.
